# Perceived Accomplishment in Later Life: The Influence of Past Decisions Between Family and Work

**DOI:** 10.1093/geroni/igab046.103

**Published:** 2021-12-17

**Authors:** Jeongsoo Park, Marina Larkina, Jacqui Smith

**Affiliations:** 1 Ajou University, Suwon-si, Kyonggi-do, Republic of Korea; 2 University of Michigan, Ann Arbor, Michigan, United States; 3 University of Michigan, University of Michigan, Michigan, United States

## Abstract

Whereas previous studies have investigated life regrets, little attention has been paid to the important accomplishments older adults include in their autobiographic life narratives. Phenomenon such as the memory positivity effect suggest that accomplishments should be observed. We used a Health and Retirement Study 2017 Life History Mail Survey (N = 2,165) to examine the characteristics of participants over age 65 who reported accomplishments (max = 3), what was reported, and whether early-life decisions about balancing family and work are associated with the reports. Women, whites, and people with at least high school education and normal cognitive status were more likely to report accomplishments (67%). We categorized reports as family-related (39%), personal (19%), combined family/personal (25%) or other (16%). Multinominal logistic regression models revealed that participants who themselves favored family over work in early life or whose spouse decided for family, were more likely to report family-related accomplishments.

